# Supplementary material for: The design, analysis and application of mouse clinical trials in oncology drug development
Source: BMC Cancer. 2019 Jul 22;19:718. doi: 10.1186/s12885-019-5907-7 (PMC6643318; doi:10.1186/s12885-019-5907-7)
Supplement: Supplementary file 1 — Figure S1. Mouse number and measurement accuracy of categorical responses defined by the RECIST criteria. Figure S2. Mouse number and measurement accuracy of categorical responses defined by the 3-cat criterion. Figure S3. Mouse number and measurement accuracy of categorical responses defined by the 5-cat criterion. Figure S4. A unique treatment model classified as PD by mRECIST method, though tumor completely disappeared at end of study. Figure S5. AUC ratio as a continuous metric for MCTs. Figure S6. (a) Distribution of coefficient of determination between log-transformed tumor volume and day for PDX mice under vehicle treatment. Figure S7. Growth curves of 42 PDXs under vehicle treatment (a) and cisplatin treatment (b). Figure S8. Fitting diagnostics of the linear mixed model in Eq. 3 for the cisplatin MCT dataset (cf. Fig. S7). Figure S9. Tumor volume doubling time in PDXs for 10 cancers. Figure S10. Growth curves of 27 PDXs under (a) vehicle treatment and (b) cetuximab treatment (1 mg/mouse, intraperitoneal injection, once per week). Figure S11. In the EXPAND phase III trial (1), for patients with IHC score greater than ~ 200, the 7 patients receiving cetuximab in addition to had significantly longer (a) PFS and (b) OS than the 19 patients receiving only chemotherapies. Figure S12. TGI is a growth rate biased and time-dependent efficacy metric. Figure S13. Growth curves of 16 PDXs under (a) vehicle treatment and (b) Irinotecan treatment (100 mg/kg, intraperitoneal injection, once per week for 2–3 weeks. Table S1. Objective response rate (ORR) in 4 categorizing methods. Table S2. Irinotecan response of 16 PDX models by 4 categorical endpoint methods. Table S3. Most enrichment pathways in Reactome 2016 database for the Irinotecan MCT. Table S4. Most enrichment terms in GO Biological Processes for the Irinotecan MCT. (PDF 2790 kb) [file 12885_2019_5907_MOESM1_ESM.pdf]

The design, analysis and application of mouse clinical trials in oncology drug development

Authors: Sheng Guo[1*], Xiaoqian Jiang[1], Binchen Mao[1], Henry Li[2,3*]

Affiliations: [1]Crown Bioscience Inc., 218 Xinghu Street, Suzhou Industrial Park, Jiangsu, China, 215028. [2]Crown Bioscience, Inc., 3375 Scott Blvd, Suite 108, Santa Clara, CA 95054, USA. [3]State Key Laboratory of Natural and Biomimetic Drugs, Peking University, Beijing, China, 100191.

*: Correspondence to guosheng@crownbio.com and henryli@crownbio.com

Running title: design, analysis and application of mouse clinical trials

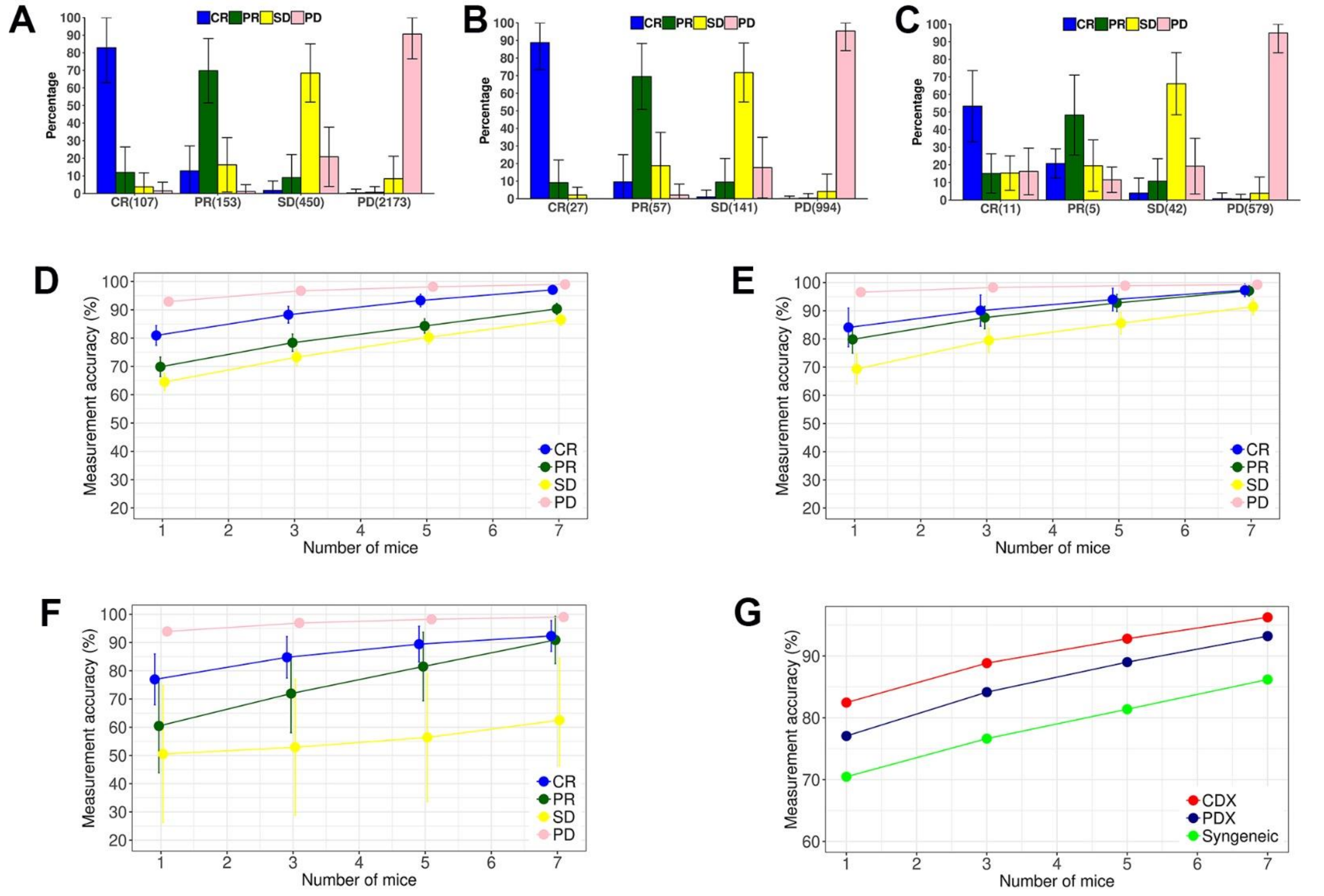

Figure S1. Mouse number and measurement accuracy of categorical responses defined by the RECIST criteria. (**a-c**): individual mouse response and majority response in PDX (**a**), CDX (**b**) and syngeneic models (**c**), x axis is the number of majority response from 4 response categories (CR: complete response, PR: partial response, SD: stable disease, PD: progressive disease.), y axis is the percentage of individual mouse response relative to the majority (average ± s.d.). There are 26,127 mice in 2,883 unique treatment PDX models, 11,139 mice in 1,219 unique treatment CDX models, and 5,945 mice in 637 unique treatment syngeneic models. Each unique treatment model had at least 8 mice. (**d-g**): measurement accuracy increases with number of mice for PDX (**d**), CDX (**e**) and syngeneic models (**f**). For each unique treatment model, the majority response of n (n=1, 3, 5, 7 in x axis) randomly sampled mice was obtained to see if it agreed with the actual majority response. The procedure was repeated 1,000 times to obtain the accuracy—percentage of times (average ± s.d.) that they agreed—for the 4 response categories, whose unweighted average is shown in (**g**).

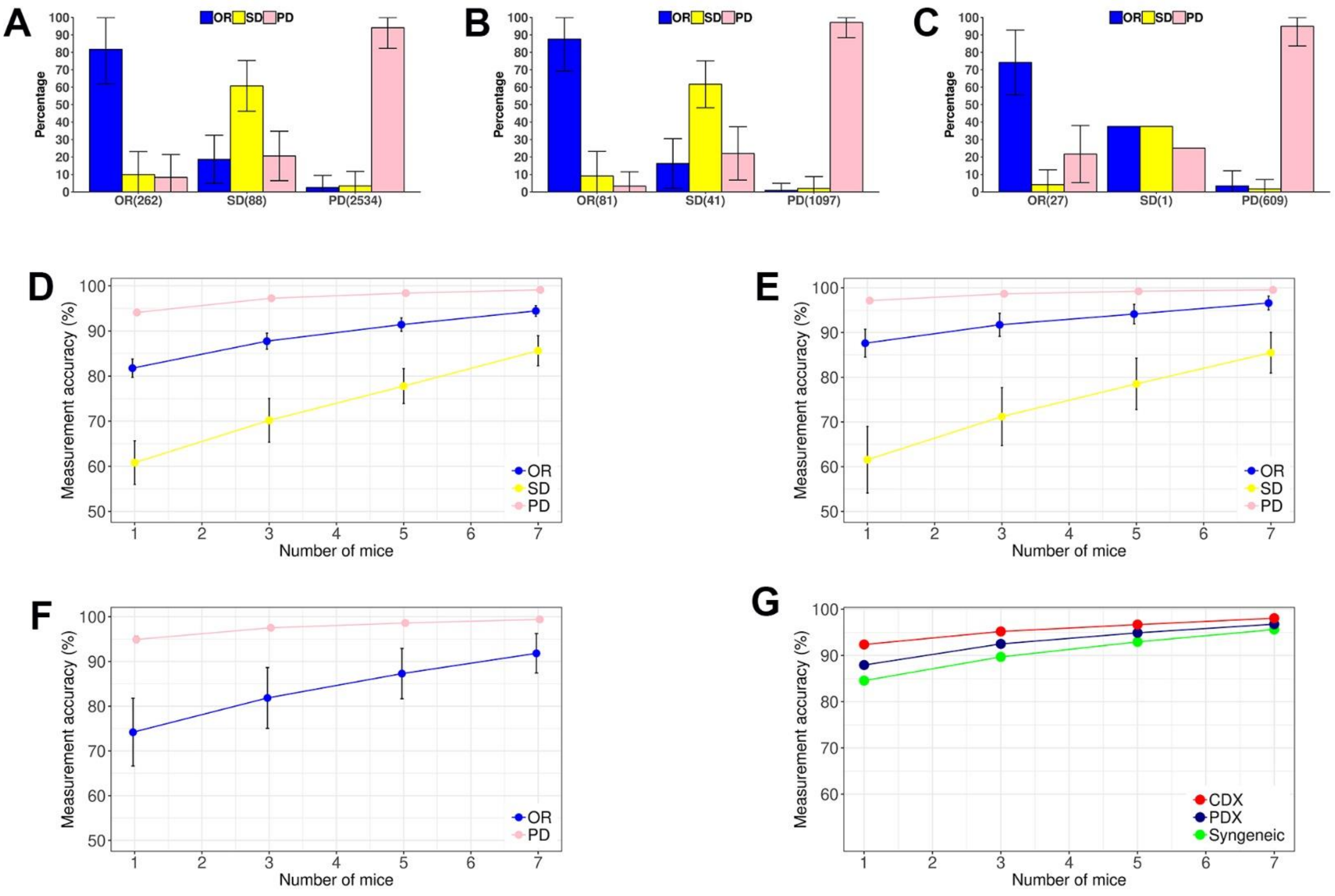

Figure S2. Mouse number and measurement accuracy of categorical responses defined by the 3-cat criteria. (**a-c**): individual mouse response and majority response in PDX (**a**), CDX (**b**) and syngeneic models (**c**), x axis is the number of majority response from 4 response categories (OR: objective response, SD: stable disease, PD: progressive disease.), y axis is the percentage of individual mouse response relative to the majority (average ± s.d.). There are 26,127 mice in 2,883 unique treatment PDX models, 11,139 mice in 1,219 unique treatment CDX models, and 5,945 mice in 637 unique treatment syngeneic models. Each unique treatment model had at least 8 mice. (**d-g**): measurement accuracy increases with number of mice for PDX (**d**), CDX (**e**) and syngeneic models (**f**). For each unique treatment model, the majority response of n (n=1, 3, 5, 7 in x axis) randomly sampled mice was obtained to see if it agreed with the actual majority response. The procedure was repeated 1,000 times to obtain the accuracy—percentage of times (average ± s.d.) that they agreed—for 2 response categories (OR and PD), whose unweighted average is shown in (**g**).

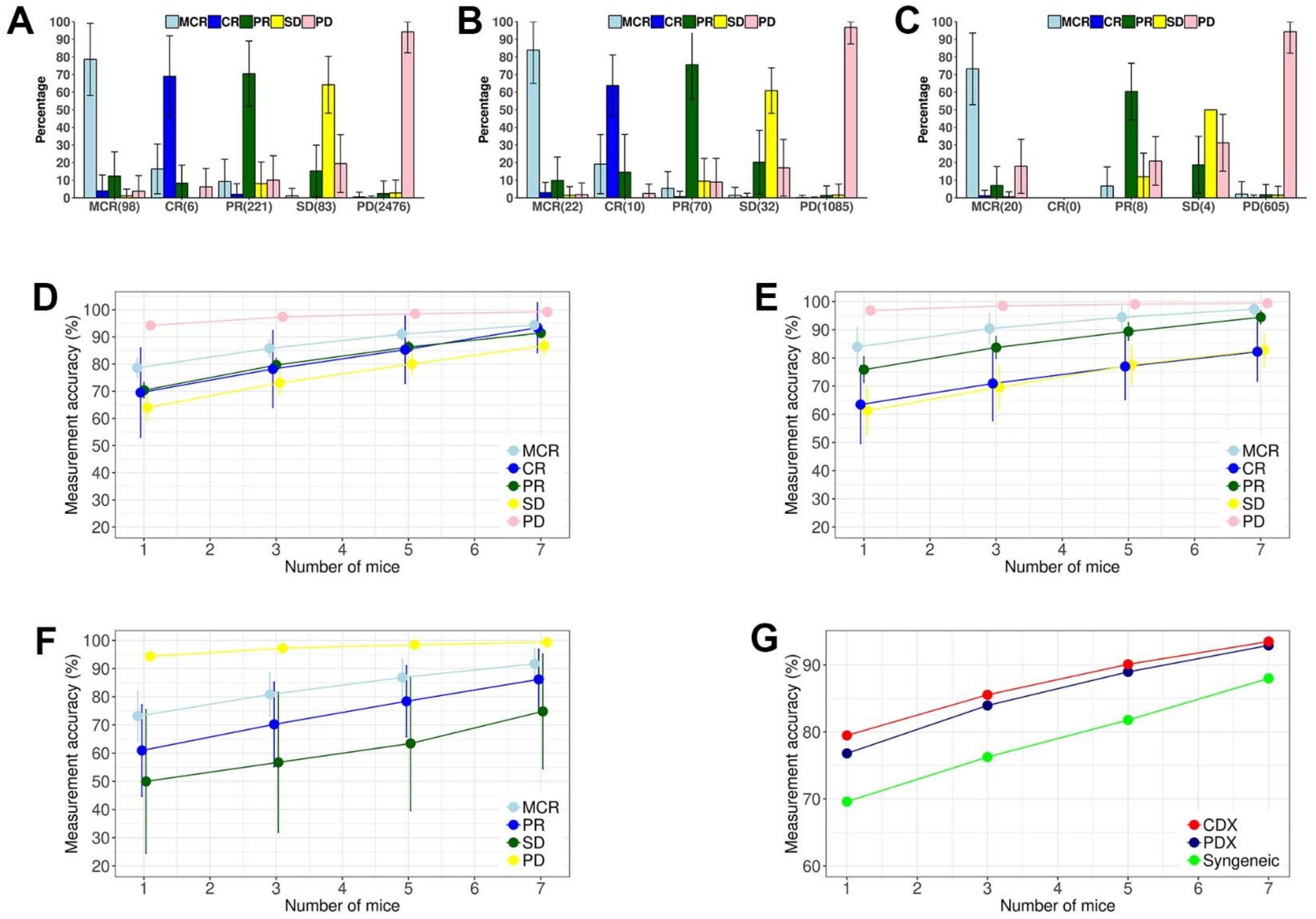

Figure S3. Mouse number and measurement accuracy of categorical responses defined by the 5-cat criteria. (**a-c**): individual mouse response and majority response in PDX (**a**), CDX (**b**) and syngeneic models (**c**), x axis is the number of majority response from 4 response categories (MCR: maintained complete response, CR: complete response, PR: partial response, SD: stable disease, PD: progressive disease.), y axis is the percentage of individual mouse response relative to the majority (average ± s.d.). There are 26,127 mice in 2,883 unique treatment PDX models, 11,139 mice in 1,219 unique treatment CDX models, and 5,945 mice in 637 unique treatment syngeneic models. Each unique treatment model had at least 8 mice. (**d-g**): measurement accuracy increases with number of mice for PDX (**d**), CDX (**e**) and syngeneic models (**f**). For each unique treatment model, the majority response of n (n=1, 3, 5, 7 in x axis) randomly sampled mice was obtained to see if it agreed with the actual majority response. The procedure was repeated 1,000 times to obtain the accuracy—percentage of times (average ± s.d.) that they agreed—for 4 response categories (excluding CR), whose unweighted average is shown in (**g**).

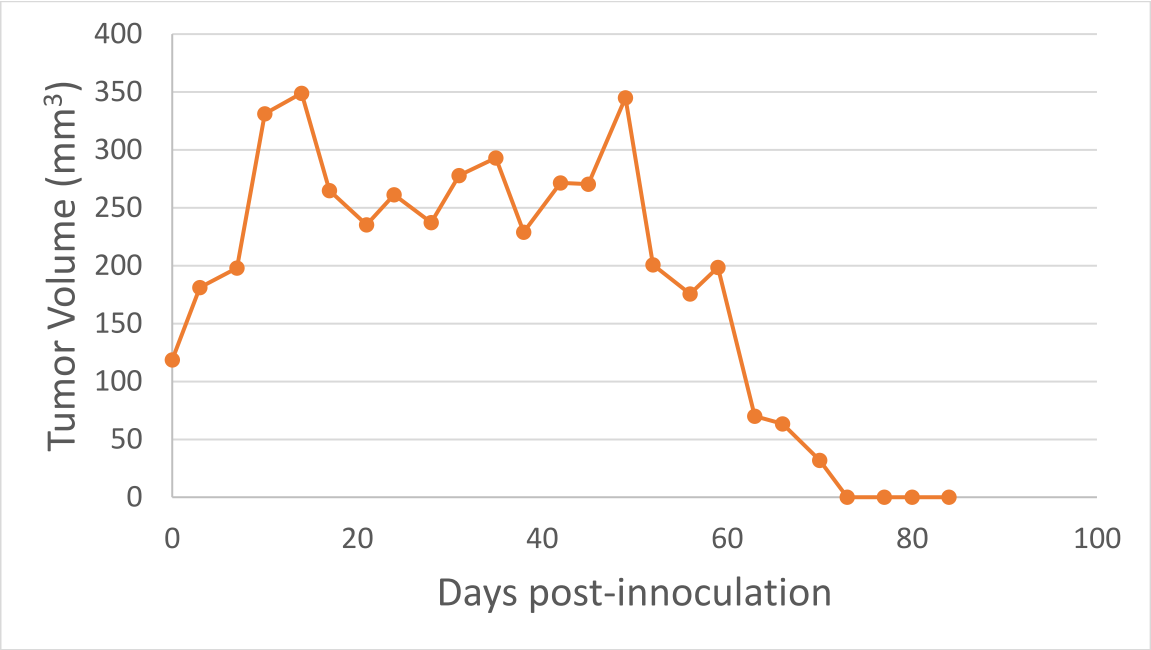

Figure S4. A unique treatment model classified as PD by the mRECIST method, even though tumor completely disappeared at end of study (BestResponse = -100%, BestAvgResponse = 59.2%)

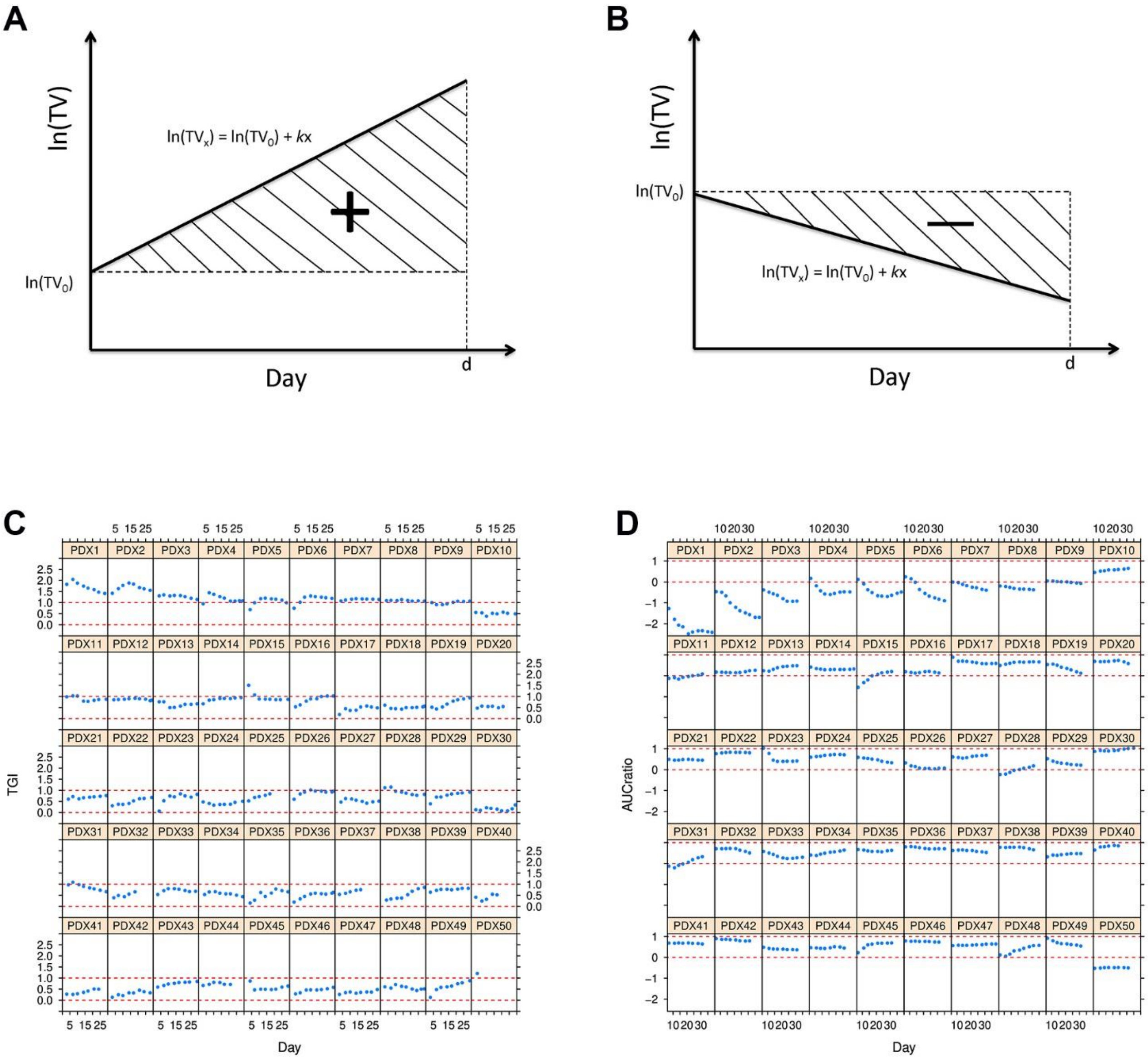

Figure S5. AUC ratio as a continuous metric for MCTs. AUC is area under curve obtained from the tumor growth data, as illustrated in (**a-b**). Assuming tumor grows with exponential kinetics (Equation 1 in Materials and Methods), k_T and k_C are the rate constants for the drug group and vehicle group, k_T/k_C is a constant measuring tumor response, hence a continuous metric. We can show that $\frac{k_T}{k_c} = \frac{\int_0^d \ln V_x^{(T)} dx - \mathrm{d}\times \ln V_0^{(T)}}{\int_0^d \ln V_x^{(C)} dx - \mathrm{d}\times \ln V_0^{(C)}} = \frac{AUC_T}{AUC_C}$, therefore, AUC ratio $\frac{AUC_T}{AUC_C}$ is also a continuous efficacy endpoint. If a study stops at different time for the two groups, d_T for the drug group and d_C for the vehicle group, we normalize the AUCs by d_T^2 and d_C^2, respectively. For convenience, we still call the normalized AUC as AUC, and the ratio between the two normalized AUCs as the AUC ratio, which equals $\frac{k_T}{k_c} = \frac{AUC_T/d_T^2}{AUC_C/d_C^2}$. A smaller AUC ratio indicates better drug efficacy. When the ratio is negative, tumor shrinks under drug treatment; when the ratio is larger than 1, tumor grows faster under drug treatment. We can use the trapezoidal rule to obtain AUCs from the growth curves, even when they are non-exponential, for which AUC is a measure of average or aggregated drug effect during study period. Similar to TGI, AUC ratio varies more in the first days before reaching stable response. Unlike TGI that approaches 1 with time, AUC ratio converges to a value, which is k_T/k_C under exponential growth kinetics, and which may be far away from 1, as shown from a MCT of 50 PDXs (**c-d, cf. Figure S12**).

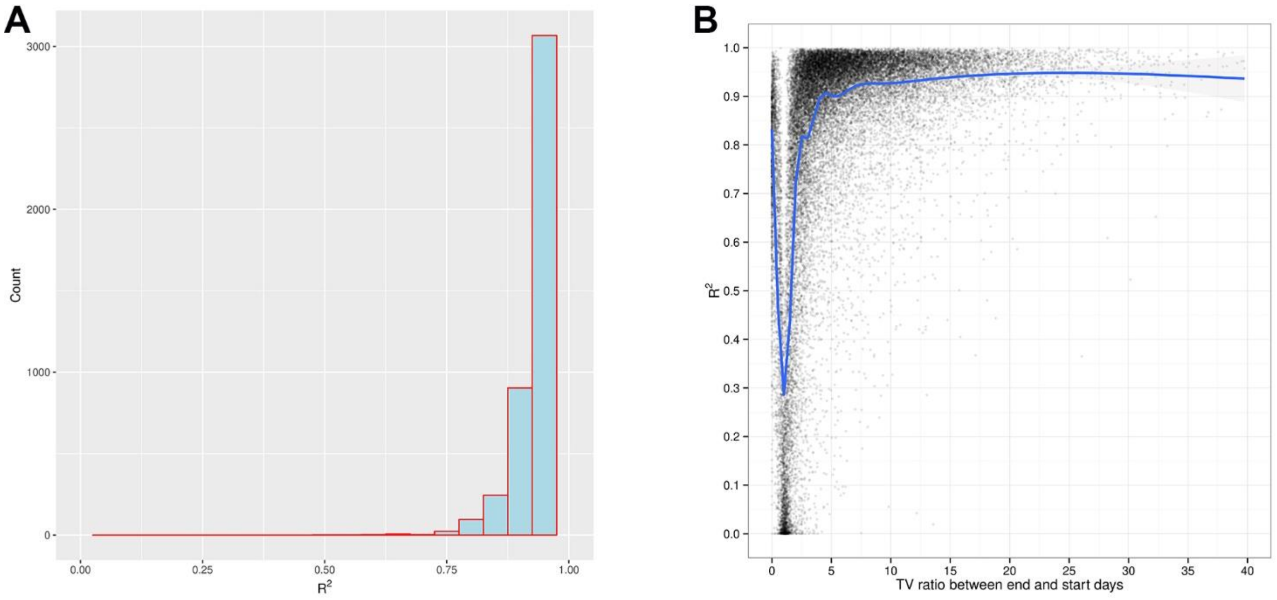

Figure S6. (**a**) Distribution of coefficient of determination (R^2) between log-transformed tumor volume and day for PDX mice under vehicle treatment. R^2 is >0.50 for nearly all mice, >0.75 for 99.7 % of mice, >0.80 for 98.9 % of mice, >0.90 for 96.5 % of mice, and >0.90 for 90 % of mice. (**b**) For 27771 mice under drug treatment, coefficient of determination (R^2) between log-transformed tumor volume and day is dependent on tumor growth, which is measured by relative tumor volume, or RTV, between ending day and starting day. R^2 is smallest when the TV ratio is around 1, at which there is, by definition, no correlation. R^2 increases quickly when RTV goes down and up. The percentage of growth curves with R^2 >0.50 is 96.7%, 98.3%, and 98.7% when RTV is larger than 2, 3, and 4, respectively. If RTV is less than 0.5, 85.7% of growth curves have R^2 >0.50. Therefore, under drug treatment, most tumors either do not grow (or shrink) much, or grow (or shrink) exponentially that can be well modelled by Equations 1 in Materials and Methods.

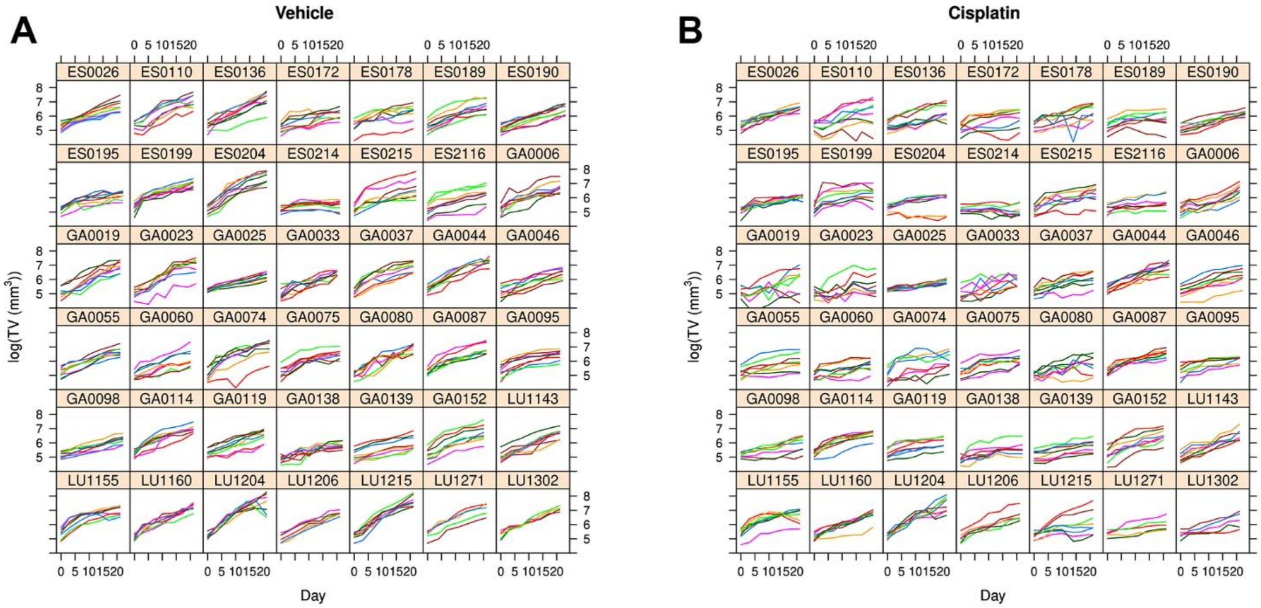

Figure S7. Growth curves of 42 PDXs under vehicle treatment (**a**) and cisplatin treatment (**b**). In the cisplatin treatment group, each mouse received cisplatin at 4mg/kg weekly for 3 weeks. There are 13 esophageal cancers (ES), 21 gastric cancers (GA) and 8 lung cancers (LU), each PDX had 5 to 9 mice.

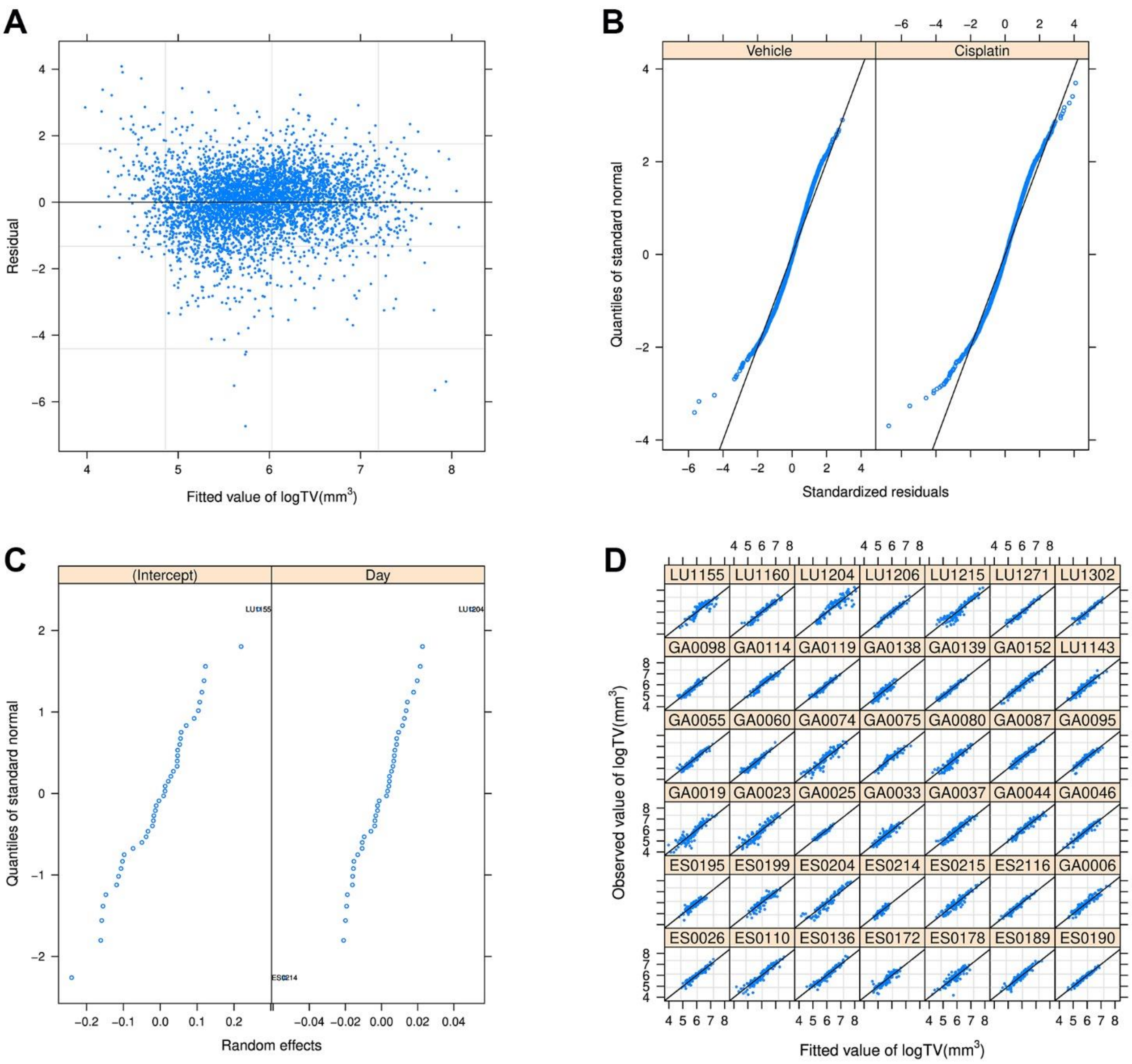

Figure S8. Fitting diagnostics of the linear mixed model in Equation 3 for the cisplatin MCT dataset (cf. Figure S7) show that the data are well fitted by the mixed model. (**a**) Plot of residuals versus fitted logTV values, showing good linearity and near constant residual variance of the data under the linear mixed model. (**b**) Normal probability plots of standardized residuals within vehicle treatment and cisplatin treatment group, showing good normality with only small numbers of deviating data points. (**c**) Normal plots of random effects, showing good fitting under the linear mixed model. (**d**) Observed logTV values versus fitted logTV values from the linear mixed model, showing good linear relationship and suggesting model fitting is proper.

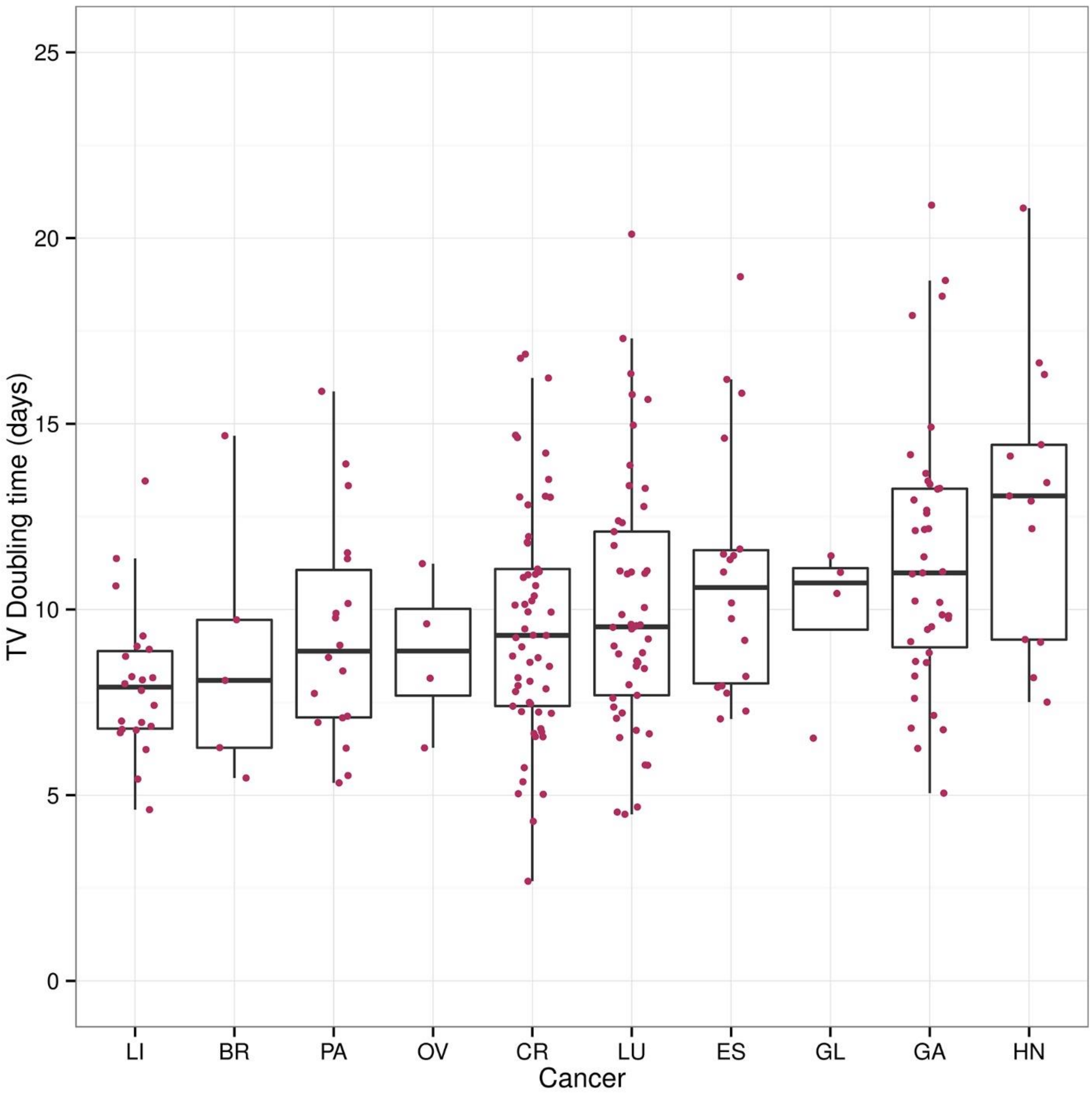

Figure S9. Tumor volume doubling time in PDXs for 10 cancers. LI: liver, BR: breast, PA: pancreatic, OV: ovarian, CR: colorectal, LU: lung, ES: esophageal, GL: gallbladder, GA: gastric, HN: head and neck.

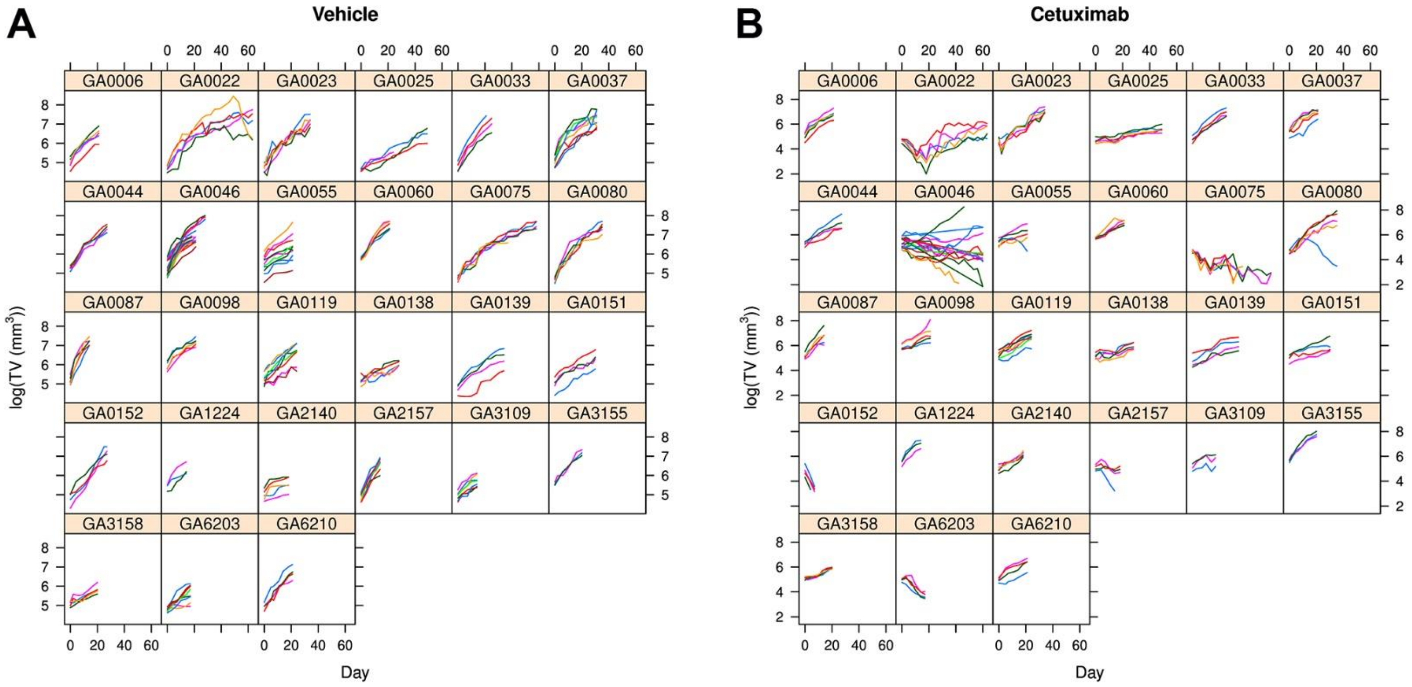

Figure S10. Growth curves of 27 PDXs under (**a**) vehicle treatment and (**b**) cetuximab treatment (1mg/mouse, intraperitoneal injection, once per week), each PDX had 3 to 10 mice.

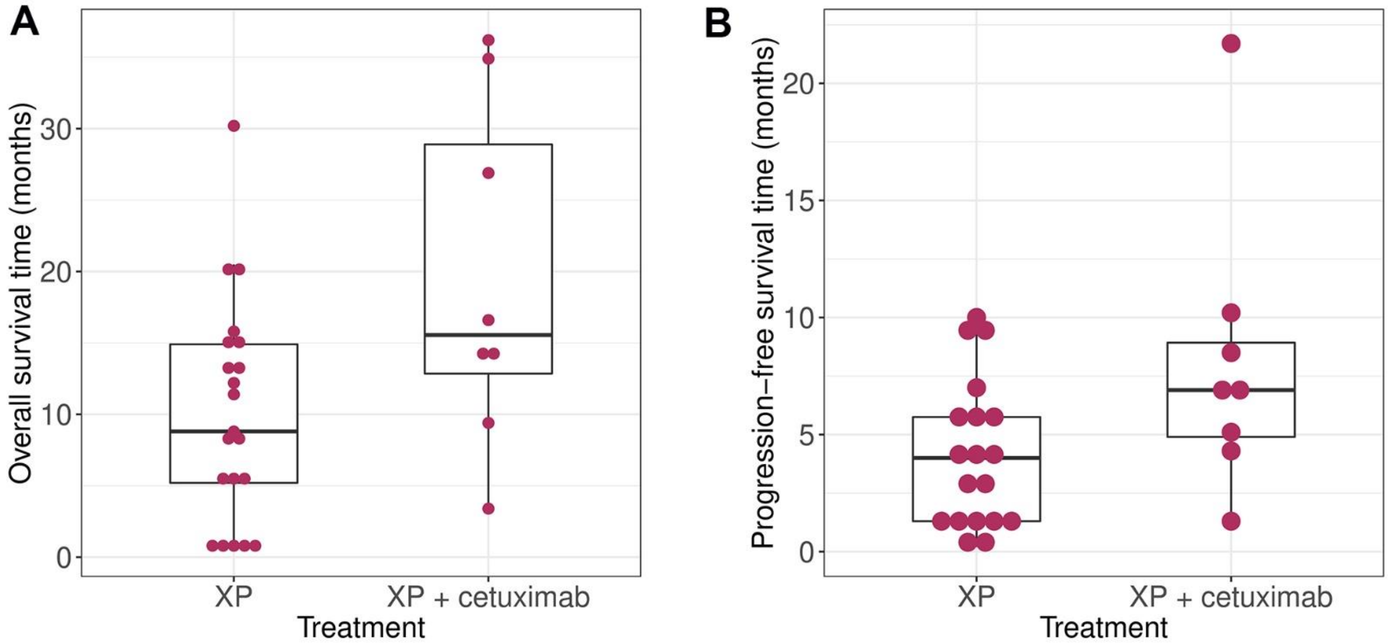

Figure S11. In the EXPAND phase III trial(1), for patients with IHC score greater than ~200, the 7 patients receiving cetuximab in addition to had significantly longer (**a**) PFS (8.1 ± 6.1 months, one-sided Mann-Whitney U-test p-value=0.03) and (**b**) OS (19.5 ± 12.0 months, p-value=0.02) than the 19 patients receiving only chemotherapies (PFS= 4.1 ± 3.2 months, OS=10.1 ± 7.8 months). Two censored patients with very low OS and PFS in the chemotherapy group were excluded from the analysis. Data were based on the supplemental Figure A1 of the trial report (1).

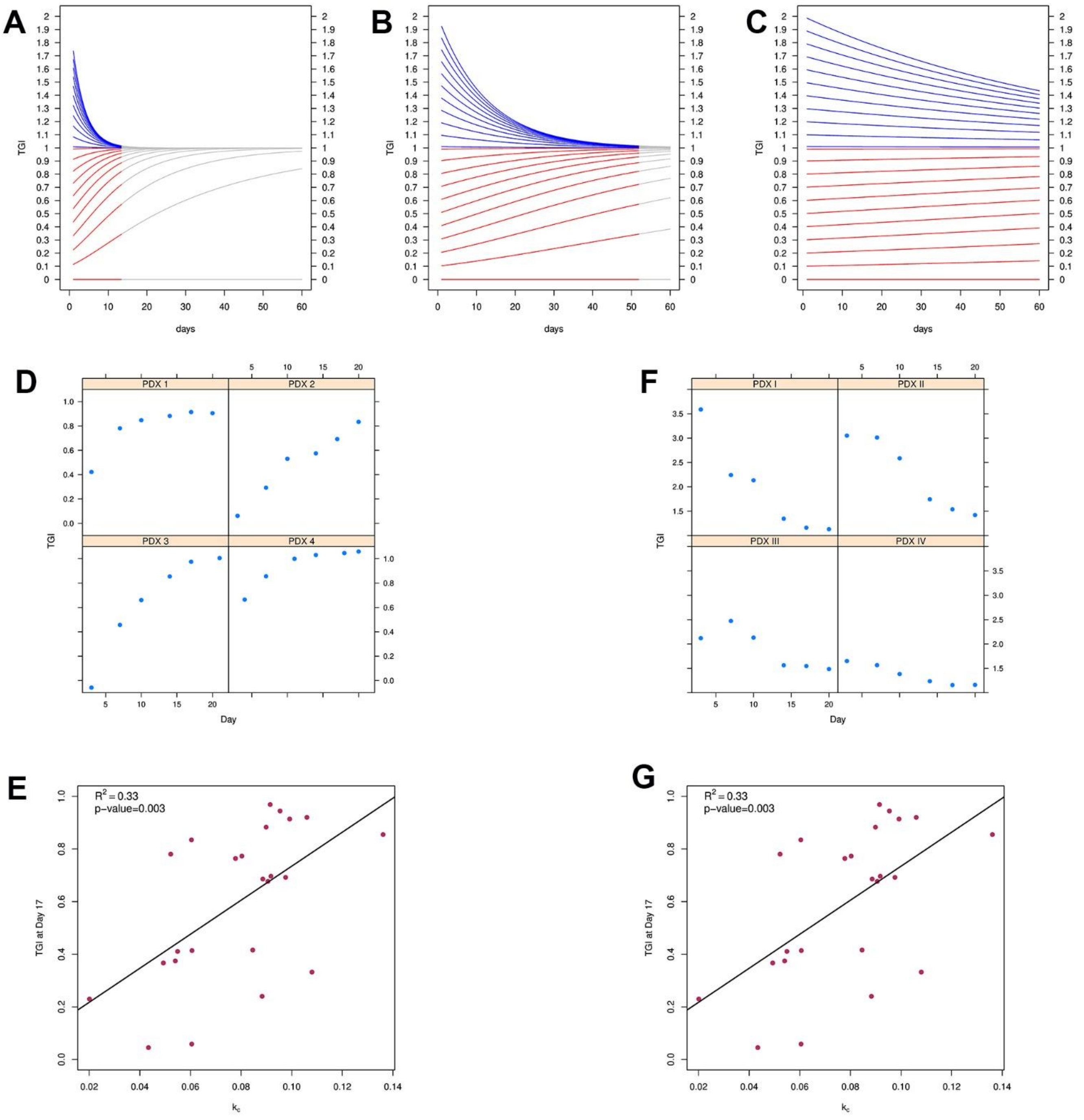

Figure S12. TGI is a growth rate biased and time-dependent efficacy metric. (**a-c**) The variation of TGI by day and by drug efficacy in PDXs with fastest (**a**), median (**b**), and slowest (**c**) growth rate, i.e. k_C, based on Equation 1 with $k_T \leq k_C$. In a graph, each line has the same k_T/k_C value. For the red lines, k_T/k_C ranges from 1 to 0 with 0.1 stepwise decrease from bottom to top. For the blue lines, k_T/k_C ranges from -1 to 0 with 0.1 stepwise increase from top to bottom. For clarify, the blue and red lines at $k_T/k_C = 0$ are drawn with slight separation. Gray portion of the lines means that the tumor volumes are larger than 3000mm^3, at which mice are sacrificed and TGIs no longer exist. (**d-e**) TGIs at 6 measurement days for the first 4 PDXs in a MCT. TGI increases by day when it is between 0 and 1. A strong positive correlation is observed between tumor growth rate k_c and TGI at day 17 for 24 PDXs whose TGIs are between 0 and 1 for all measurement days. (**f-g**) TGIs at 6 measurement days for the first 4 PDXs in a second MCT. TGI decreases by day when it is greater than 1. A strong negative correlation is observed between tumor growth rate k_c and TGI at day 17 for 18 PDXs whose TGIs are greater than 1 at all measurement days.

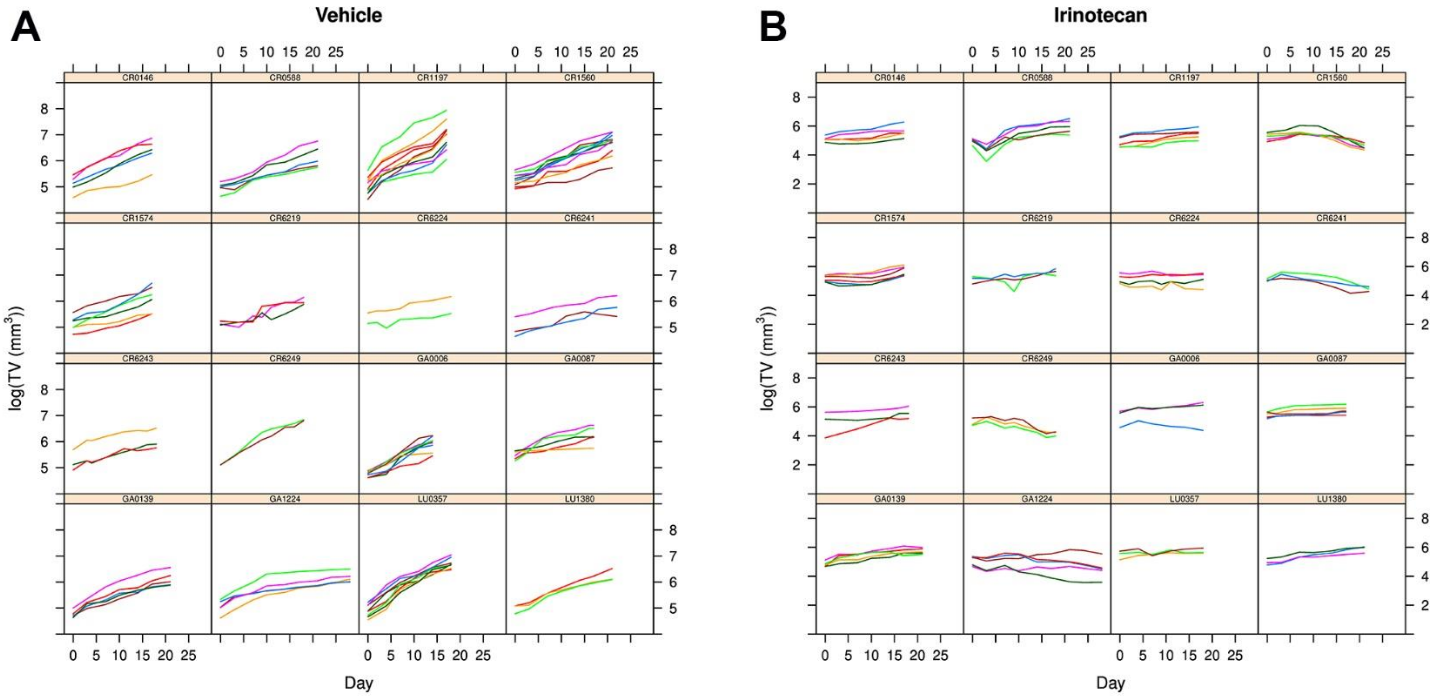

Figure S13. Growth curves of 16 PDXs under (**a**) vehicle treatment and (**b**) Irinotecan treatment (100mg/kg, intraperitoneal injection, once per week for 2-3 weeks, each PDX had 2 to 10 mice.

Supplemental Tables

Table S1. Objective response rate (ORR) in 4 categorizing methods

	PDX	CDX	Syngeneic
mRECIST	9.02%	6.89%	2.35%
RECIST	8.63%	6.40%	4.20%
3-cat	9.08%	6.64%	4.24%
5-cat	11.27%	8.37%	4.40%

Table S2. Irinotecan response of 16 PDX models by 4 categorical endpoint methods

PDX	3-cat	RECIST	mRECIST	5-cat
CR6241	OR	PR	SD	PR
CR6249	OR	PR	SD	PR
GA1224	OR	PR	SD	PR
CR1560	OR	PR	SD	SD
CR1197	PD	PD	PD	PD
GA0139	PD	PD	PD	PD
LU1380	PD	PD	PD	PD
GA0087	PD	SD	PD	PD
CR6219	PD	PD	SD	PD
GA0006	PD	PD	SD	PD
CR0146	PD	SD	SD	PD
CR1574	PD	SD	SD	PD
CR6243	PD	SD	SD	PD
CR0588	PD	PD	PD	PR
LU0357	SD	SD	SD	PD
CR6224	SD	SD	SD	SD

OR: objective response, PR: partial response, SD: stable disease, PD: progressive disease

Table S3. Most enrichment pathways in Reactome 2016 database for the Irinotecan MCT

Method	#Genes	P-value	Adjusted p-value	Log(P-value)	log(adjusted p-value)	Most enriched pathways in REACTOME2016
LMM	100	1.37E-07	4.64E-05	6.86	4.33	Cell Cycle_Homo sapiens_R-HSA-1640170
LMM	200	9.75E-11	4.39E-08	10.01	7.36	Cell Cycle_Homo sapiens_R-HSA-1640170
LMM	300	1.33E-12	7.26E-10	11.88	9.14	Cell Cycle_Homo sapiens_R-HSA-1640170
LMM	400	2.95E-20	1.98E-17	19.53	16.70	Cell Cycle_Homo sapiens_R-HSA-1640170
LMM	500	1.15E-18	8.54E-16	17.94	15.07	Cell Cycle_Homo sapiens_R-HSA-1640170
LMM	600	4.19E-20	3.33E-17	19.38	16.48	Cell Cycle_Homo sapiens_R-HSA-1640170
LMM	700	1.27E-21	1.11E-18	20.89	17.95	Cell Cycle_Homo sapiens_R-HSA-1640170
LMM	800	1.85E-26	1.72E-23	25.73	22.76	Cell Cycle_Homo sapiens_R-HSA-1640170
LMM	900	2.20E-29	2.17E-26	28.66	25.66	Cell Cycle_Homo sapiens_R-HSA-1640170
LMM	1000	1.06E-29	1.11E-26	28.97	25.96	Cell Cycle_Homo sapiens_R-HSA-1640170
LMM	1250	2.48E-35	2.75E-32	34.61	31.56	Cell Cycle_Homo sapiens_R-HSA-1640170
LMM	1500	2.85E-39	3.25E-36	38.55	35.49	Cell Cycle_Homo sapiens_R-HSA-1640170
LMM	2000	2.60E-37	3.11E-34	36.59	33.51	Cell Cycle_Homo sapiens_R-HSA-1640170
TGI	100	4.87E-06	9.69E-04	5.31	3.01	Activation of ATR in response to replication stress_Homo sapiens_R-HSA-176187
TGI	200	1.29E-08	6.84E-06	7.89	5.16	Cell Cycle_Homo sapiens_R-HSA-1640170
TGI	300	1.33E-07	8.22E-05	6.88	4.09	Cell Cycle_Homo sapiens_R-HSA-1640170
TGI	400	1.44E-07	0.00007949	6.84	4.10	Cell Cycle_Homo sapiens_R-HSA-1640170
TGI	500	5.98E-06	2.54E-03	5.22	2.59	Cell Cycle, Mitotic_Homo sapiens_R-HSA-69278
TGI	600	2.00E-07	1.76E-04	6.70	3.76	Cell Cycle_Homo sapiens_R-HSA-1640170
TGI	700	1.11E-07	1.00E-04	6.95	4.00	Cell Cycle_Homo sapiens_R-HSA-1640170
TGI	800	4.48E-06	3.54E-03	5.35	2.45	Cell Cycle_Homo sapiens_R-HSA-1640170
TGI	900	9.01E-07	8.86E-04	6.05	3.05	Cell Cycle_Homo sapiens_R-HSA-1640170
TGI	1000	1.80E-05	1.70E-02	4.74	1.77	Cell Cycle_Homo sapiens_R-HSA-1640170
TGI	1250	1.19E-05	6.40E-03	4.93	2.19	Cell Cycle, Mitotic_Homo sapiens_R-HSA-69278
TGI	1500	4.87E-07	5.45E-04	6.31	3.26	Cell Cycle_Homo sapiens_R-HSA-1640170
TGI	2000	2.93E-07	2.16E-04	6.53	3.67	Cell Cycle_Homo sapiens_R-HSA-1640170
RECIST	100	3.34E-06	1.10E-03	5.48	2.96	Cell Cycle, Mitotic_Homo sapiens_R-HSA-69278
RECIST	200	2.64E-07	1.51E-04	6.58	3.82	Cell Cycle_Homo sapiens_R-HSA-1640170
RECIST	300	2.34E-09	1.62E-06	8.63	5.79	Cell Cycle_Homo sapiens_R-HSA-1640170
RECIST	400	1.44E-07	8.62E-05	6.84	4.06	Cell Cycle_Homo sapiens_R-HSA-1640170
RECIST	500	1.22E-08	6.71E-06	7.91	5.17	Cell Cycle_Homo sapiens_R-HSA-1640170
RECIST	600	5.34E-07	1.59E-04	6.27	3.80	DNA replication-independent nucleosome assembly (GO:0006336)
RECIST	700	4.18E-06	9.79E-04	5.38	3.01	Resolution of Sister Chromatid Cohesion_Homo sapiens_R-HSA-2500257
RECIST	800	1.01E-07	5.03E-05	7.00	4.30	Cell Cycle, Mitotic_Homo sapiens_R-HSA-69278
RECIST	900	3.32E-09	1.72E-06	8.48	5.76	Cell Cycle, Mitotic_Homo sapiens_R-HSA-69278
RECIST	1000	5.56E-09	1.98E-06	8.26	5.70	Cell Cycle, Mitotic_Homo sapiens_R-HSA-69278
RECIST	1250	2.87E-09	1.61E-06	8.54	5.79	DNA replication-independent nucleosome assembly (GO:0006336)
RECIST	1500	5.16E-10	3.09E-07	9.29	6.51	DNA replication-independent nucleosome assembly (GO:0006336)
RECIST	2000	5.76E-10	3.73E-07	9.24	6.43	DNA replication (GO:0006260)

Table S4. Most enrichment terms in GO Biological Processes for the Irinotecan MCT

Method	#Genes	P-value	Adjusted p-value	Log(P-value)	log(adjusted p-value)	Most enriched GO term
LMM	100	0.000006866	0.004737	5.16	2.32	DNA replication initiation (GO:0006270)
LMM	200	8.21E-07	0.0002779	6.09	3.56	DNA replication initiation (GO:0006270)
LMM	300	2.82E-07	0.0003922	6.55	3.41	DNA replication initiation (GO:0006270)
LMM	400	3.09E-10	5.37E-07	9.51	6.27	DNA replication initiation (GO:0006270)
LMM	500	1.70E-10	3.30E-07	9.77	6.48	DNA replication initiation (GO:0006270)
LMM	600	7.64E-11	1.70E-07	10.12	6.77	DNA replication initiation (GO:0006270)
LMM	700	1.25E-09	0.000002067	8.90	5.68	DNA replication initiation (GO:0006270)
LMM	800	1.07E-11	1.37E-08	10.97	7.86	DNA replication initiation (GO:0006270)
LMM	900	7.95E-14	1.48E-10	13.10	9.83	DNA replication initiation (GO:0006270)
LMM	1000	6.69E-17	1.92E-13	16.17	12.72	DNA replication initiation (GO:0006270)
LMM	1250	1.79E-16	2.82E-13	15.75	12.55	DNA replication initiation (GO:0006270)
LMM	1500	2.63E-16	4.40E-13	15.58	12.36	DNA replication initiation (GO:0006270)
LMM	2000	1.94E-16	2.39E-13	15.71	12.62	DNA replication initiation (GO:0006270)
TGI	100	6.89E-03	2.91E-01	2.16	0.54	cell cycle G1/S phase transition (GO:0044843)
TGI	200	1.93E-03	1.15E-01	2.72	0.94	mitotic cell cycle phase transition (GO:0044772)
TGI	300	4.90E-04	5.09E-02	3.31	1.29	mitotic cell cycle phase transition (GO:0044772)
TGI	400	0.001429	0.1489	2.84	0.83	mitotic cell cycle phase transition (GO:0044772)
TGI	500	3.22E-03	3.39E-01	2.49	0.47	mitotic sister chromatid segregation (GO:0000070)
TGI	600	7.71E-04	1.34E-01	3.11	0.87	mitotic cell cycle phase transition (GO:0044772)
TGI	700	1.80E-04	3.38E-02	3.74	1.47	mitotic cell cycle phase transition (GO:0044772)
TGI	800	3.71E-04	6.98E-02	3.43	1.16	DNA replication (GO:0006260)
TGI	900	6.93E-04	9.19E-02	3.16	1.04	double-strand break repair via homologous recombination (GO:0000724)
TGI	1000	1.20E-03	1.55E-01	2.92	0.81	double-strand break repair via homologous recombination (GO:0000724)
TGI	1250	0.003727	0.3354	2.43	0.47	mitotic cell cycle phase transition (GO:0044772)
TGI	1500	0.00004966	0.03284	4.30	1.48	mitotic cell cycle phase transition (GO:0044772)
TGI	2000	0.0003384	0.1574	3.47	0.80	mitotic cell cycle phase transition (GO:0044772)
Endpoint	100	0.000002153	0.00195	5.67	2.71	establishment of mitotic spindle localization (GO:0040001)
Endpoint	200	0.00003344	0.04156	4.48	1.38	establishment of mitotic spindle localization (GO:0040001)
Endpoint	300	0.00003474	0.05701	4.46	1.24	mitotic sister chromatid segregation (GO:0000070)
Endpoint	400	0.0000288	0.02616	4.54	1.58	establishment of mitotic spindle localization (GO:0040001)
Endpoint	500	3.77E-08	0.00007807	7.42	4.11	DNA replication-independent nucleosome assembly (GO:0006336)
Endpoint	600	2.06E-07	0.0004816	6.69	3.32	DNA replication-independent nucleosome assembly (GO:0006336)
Endpoint	700	8.47E-07	0.002138	6.07	2.67	DNA replication-independent nucleosome assembly (GO:0006336)
Endpoint	800	2.82E-06	0.007608	5.55	2.12	DNA replication-independent nucleosome assembly (GO:0006336)
Endpoint	900	1.00E-06	0.002879	6.00	2.54	DNA replication-independent nucleosome assembly (GO:0006336)
Endpoint	1000	2.80E-06	0.00838	5.55	2.08	DNA replication-independent nucleosome assembly (GO:0006336)
Endpoint	1250	3.66E-05	0.01212	4.44	1.92	DNA replication-independent nucleosome assembly (GO:0006336)
Endpoint	1500	6.07E-07	0.00216	6.22	2.67	DNA replication-independent nucleosome assembly (GO:0006336)
Endpoint	2000	2.09E-06	0.006681	5.68	2.18	DNA replication (GO:0006260)

References

(1) Lordick, F., Kang, Y. K., Chung, H. C., Salman, P., Oh, S. C., Bodoky, G., Kurteva, G., Volovat, C., Moiseyenko, V. M., Gorbunova, V., et al. (2013). Capecitabine and cisplatin with or without cetuximab for patients with previously untreated advanced gastric cancer (EXPAND): a randomised, open-label phase 3 trial. Lancet Oncol 14, 490-499.
